# A trial design for evaluation of empiric programming of implantable cardioverter defibrillators to improve patient management

**DOI:** 10.1186/1468-6708-5-12

**Published:** 2004-11-12

**Authors:** John M Morgan, Laurence D Sterns, Jodi L Hanson, Kevin T Ousdigian, Mary F Otterness, Bruce L Wilkoff

**Affiliations:** 1 Southampton General Hospital, Southampton, United Kingdom; 2 Victoria Heart Institute, Victoria, BC, Canada; 3 Medtronic, Inc., Minneapolis, MN, United States; 4 Cleveland Clinic Foundation, Cleveland, OH, United States

**Keywords:** Implantable cardioverter defibrillator, ICD, shocks, programming, empiric, detection, therapy

## Abstract

The delivery of implantable cardioverter defibrillator (ICD) therapy is sophisticated and requires the programming of over 100 settings. Physicians tailor these settings with the intention of optimizing ICD therapeutic efficacy, but the usefulness of this approach has not been studied and is unknown. Empiric programming of settings such as anti-tachycardia pacing (ATP) has been demonstrated to be effective, but an empiric approach to programming all VT/VF detection and therapy settings has not been studied. A single standardized empiric programming regimen was developed based on key strategies with the intention of restricting shock delivery to circumstances when it is the only effective and appropriate therapy. The EMPIRIC trial is a worldwide, multi-center, prospective, one-to-one randomized comparison of empiric to physician tailored programming for VT/VF detection and therapy in a broad group of about 900 dual chamber ICD patients. The trial will provide a better understanding of how particular programming strategies impact the quantity of shocks delivered and facilitate optimization of complex ICD programming.

## Background

Over the past decade ICD implantation has become increasingly straightforward, yet ICD programming and follow up has become more complex due to device feature and capability enhancements. While sophisticated algorithms provide high sensitivity and improved specificity of arrhythmia detection, allowing delivery of necessary effective therapy with minimization of inappropriate defibrillation shocks, detection and therapy of ventricular tachycardia (VT) / ventricular fibrillation (VF) still requires programming about 100 settings [1-3].

Good programming choices are crucial as they relate to patient acceptance of ICD therapy. It has been found that patients who receive multiple shocks have greater difficulty adjusting to the ICD implant. These patients may become anxious or depressed, especially if a prior history of these ailments exists [4]. Reducing shocks delivered to the patient would improve overall patient management.

To date, there is no proven consensus on how to use information about the patient's complex diseases to program the ICD, and usually little is known about the patient's spontaneous VT rates, their risk of syncope, or therapies to effectively terminate spontaneous ventricular arrhythmias. Furthermore, ICD indications have dramatically changed within the last five years. Physicians may retain old programming habits even with enhanced devices or expanding patient indications, which may result in sub-optimal detection and therapy, such as unnecessary shocks for faster VT, supraventricular tachycardia (SVT), and non-sustained VT. Physicians often adjust many programmable settings that may benefit the patient. For example, physicians may prescribe patient-specific regimens for anti-tachycardia pacing (ATP) or shock energies based on lab testing. While one would expect this tailoring of programming to improve outcomes, it has never been studied.

Empiric programming has been shown to be effective for subsets of ICD settings, including subsets of dual chamber detection and ATP therapies [3,5-10]. Whether this holds true for comprehensive programming of VT/VF detection and therapy for all ICD patients is unknown.

A proven optimal programming approach would be useful for simplifying therapy prescription, improving therapy outcomes, reducing inadvertent programming errors, and overall reducing shock-related morbidity. The EMPIRIC trial has been designed to evaluate a standardized empiric programming regimen by testing the hypothesis stated below. The EMPIRIC trial outcome will provide an understanding of how programming strategies impact defibrillation shock delivery in ICD therapy.

## EMPIRIC Trial Hypothesis

This trial tests the hypothesis that the shock related morbidity of ICD therapy is similar whether patients are treated with a standardized empiric programming regimen for VT/VF detection and therapy or with a patient-specific physician tailored approach.

### Indices of Shock Morbidity

Only sustained VT/VF that cannot be painlessly terminated should result in shock therapy and it is unusual for supraventricular arrhythmias (SVT) to require shock therapy. Shock morbidity is related to the number and frequency of shocks that patients receive and therefore morbidity is reduced if shocks are delivered only when necessary for effective arrhythmia termination. Thus, indices that address shock morbidity should reflect both the frequency and appropriateness of shocks for VT/VF and SVT.

Shock morbidity is quantifiable by determination of the following:

♦ proportion of ***true ***VT/VF episodes that are shocked

♦ proportion of ***true ***SVT episodes that are shocked

♦ time to first shock (VT/VF or SVT)

♦ time to first VT/VF shock

♦ time to first SVT shock

These parameters are used to define the Empiric Trial's main objectives.

### Empiric Trial Objectives

The primary objective is to demonstrate that the proportion of shocked VT/VF episodes *and *the proportion of shocked SVT episodes in a population whose ICDs are programmed using a standardized regimen for VT/VF detection and therapy, is either similar to or less than the same proportion in a similar population whose ICDs are programmed using a physician-tailored approach. This primary objective was chosen to independently evaluate the effects of programming on both appropriate and inappropriate ICD shocks (which are likely to have different implications for patient management). The advantage of this approach is that it focuses on frequency of shock delivery while also allowing an assessment of their appropriateness. However, this assessment could be confounded by a disproportionate number of SVT events in the two study groups. For example, an abundance of non-shocked SVT events in the physician-tailored arm, despite a greater incidence of inappropriate SVT shock therapies in that arm, nevertheless would result in the proportion of SVT episodes shocked being similar in the two arms. The analysis is also heavily dependent on the electrogram data stored in the ICDs. Given the electrogram storage capability of ICDs, differing rates of electrogram storage might occur between study arms or between VT/VF and SVT episodes that may skew the amount of data available for analysis. Therefore, the key secondary endpoint in this study is considered to be the time to delivery of first shock therapy in any given patient. This endpoint offers the advantage that it enables patient cross over to occur between the study arms without endpoint compromise and it is a clinically robust indicator of patient shock-related morbidity. Furthermore, its analysis is not influenced by the appropriateness or otherwise of a shock therapy and therefore cannot be confounded by differential occurrence of non-shocked SVT events in the study arms.

Other secondary endpoints will further evaluate the impact of the standardized programming regimen on patients by an assessment of detection performance, health care utilization, shock impact on device longevity, and "true VT/VF" episode durations.

## EMPIRIC Trial Protocol Design

The EMPIRIC trial is a worldwide, multi-center, prospective, one-to-one randomized comparison of empiric to physician tailored programming. About 900 patients were enrolled worldwide at 52 centers from August 2002 to October 2003. Each patient will be followed for approximately one year.

The inclusion criteria require patients to meet all of the following conditions:

1. Indicated for an ICD according to internationally accepted criteria.

2. Willing to sign informed consent or offer a legal representative who can provide consent.

3. Achieved a 10 Joule safety margin at implant.

Patients are excluded if they:

1. Have permanent atrial fibrillation (AF).

2. Had a previous ICD.

3. Have a medical condition that precludes the testing required by the protocol or limited trial participation.

4. Have a life expectancy less than one year.

5. Are unable to complete follow-ups at the trial center.

6. Are enrolled or participating in another clinical trial.

### Randomization

Patients receiving a Marquis DR ICD are randomized to one of the two programming approaches after meeting a 10 J safety margin. In order to control for physician practice between the two treatment arms, randomization is stratified by treatment center. Further, since the incidence and prevalence of spontaneous VT/VF and SVT among primary prevention patients is not well known, randomization is also stratified by ICD indication (secondary vs. primary). A secondary indication includes patients with a history of spontaneous sustained VT/VF or syncope with suspected VT. A primary prevention indication includes all other patients.

### Programming Approaches

The physician tailored approach is based on the standard practice of each physician. All VT/VF programming may be tailored to the patient except that VT detection must be turned to 'On' or 'Monitor' to record episodes of slower VT.

The empiric standardized regimen is based on various programming strategies to reduce shocks. In this arm, initial device settings are fixed (see Table 1), with the exception of the VT detection interval, which can be set slower than 150 bpm when clinically necessary.

**Table 1:** Empiric Arm Programming

**Detection**		**Interval**	**Beats To Detect **	**Redetect**	**Therapies**
VF	On	300 ms	18/24 9/12	9/12	30 J × 6
FVT	via VF	240 ms	NA		Burst (1 sequence), 30 J × 5
VT	On	≥ 400 ms*	16	12	Burst (2), Ramp (1), 20 J, 30 J × 3

SVT Criteria On: AF/Afl, Sinus Tach (1:1 VT-ST Boundary = 66%), SVT Limit = 300 ms

Burst ATP: 8 intervals, R-S1 = 88%, 20 ms decrement

Ramp ATP: 8 intervals, R-S1 = 81%, 10 ms decrement

VT/VF detection and therapy programming changes are permitted at follow-up in both arms only when medically justified. These changes must be documented, and are reviewed throughout the study.

### Data Collection

Patients are followed for a 12-month period, with required clinic visits at 3, 6 and 12 months. Data collection includes: VT/VF and SVT episodes, device programming, medical justifications for VT/VF programming changes, cardiovascular medication, adverse device events, P and R wave measurements, and cardiovascular-related hospitalizations.

### Study Design Challenges

A challenge of the study design is the possibility that physician practice could become biased by in-trial experience, causing physician practice to gravitate towards the empiric standardized regimen. This might occur if empiric programming is perceived to be efficacious, particularly with respect to management of rapid ventricular tachycardia by pace termination. Collection of pre-trial programming practices provides the capacity to evaluate potential "treatment drift". This result will be reported. Additionally, in an effort to prevent drifting or possible physician bias to programming in the physician tailored arm, a weekly comparison of programming status and initial implant programming will be assessed through device interrogation information. Any programming changes made must be supported by a medical justification with a basis of event-related occurrences (i.e. system- or procedure-related adverse events, spontaneous episodes, or inappropriate shocks). In order to protect protocol design integrity, reprogramming will be encouraged for non-justified programming deviations. In this manner the initial treatment strategies are tested using an intention-to-treat analysis with characterization of programming changes.

## Empiric Arm Programming Strategies

The empiric arm standardized programming regimen is based on the following key strategies to reduce shocks.

### 1) Strategies to reduce shocks for VT/VF

• ***Multiple ATP attempts for VT≤ 200 bpm: ***Three sequences of ATP will be attempted for rhythms with ventricular rates ≤ 200 bpm. Empiric ATP has been shown to terminate ≥ 90% of VTs in the VT zone [5-10]. Furthermore, induced VTs do not predict spontaneous VT cycle length, morphology, or therapy efficacy [11]. Three sequences will be attempted for rates up to 200 bpm because the average rate of fast VTs was 199 bpm in the PainFREE Rx1 study, where the FVT zone was 188 – 250 bpm, and more ATP provided incremental shock reductions[6]. ATP will be used in all patients because even cardiac arrest patients have been shown to have VTs [5,12-14].

• ***ATP for VTs 201 – 250 bpm: ***One sequence of ATP will be delivered for fast VTs (FVT) using the FVT via VF zone, which maintains sensitivity to polymorphic VT (PVT) and VF and delivers ATP if the 8 beats prior to FVT detection are ≤ 250 bpm. Approximately 81% of ICD detected VF is monomorphic VT (MVT). MVT can be pace-terminated approximately 75% of the time with one sequence of ATP, without increased risk of syncope or acceleration [6,7,15].

• ***Longer detection duration: ***The VF initial beats to detect will be set to 18 of 24. Shorter beats to detect are often programmed by physicians, but may increase the unnecessary shocks for non-sustained VT and for SVTs. At least 25% of ICD-detected VF is non-sustained VT/VF [15-17].

• ***High Output 1^st ^VF and FVT Shock: ***A 30 Joule energy will be used for the first VF and FVT shock. This will allow additional time for spontaneous conversions that frequently occur. A higher shock energy may also improve 1^st ^shock success and therefore reduce the need for multiple shocks within an episode. The LESS study found no difference in 1^st ^shock success with 31 J versus DFT++, however it analyzed all VT/VF faster than 200 bpm [18] ATP should terminate a majority of these rhythms and for that reason the benefit of empiric high-energy shocks for polymorphic VT (PVT)/VF or after a failed ATP is unknown. The primary reason some physicians program lower energy 1^st ^shocks is due to concerns about syncope. Several recent studies have shown very low syncope rates [6,19] Furthermore, charge times are much faster and more stable over the life of the device than in older ICDs. For instance, the Medtronic Marquis DR 30 Joule charge time is 5.9 and 7.5 seconds at beginning and end of life, respectively [20].

### 2) Strategies to Reduce Shocks for SVTs and Sensing Issues

• ***Empiric SVT Criteria: ***The PR logic criteria of AF/A. Flutter and Sinus Tach will be programmed 'On' in all patients. These criteria have been shown to have a relative VT/VF sensitivity of 100% and a positive predictive value to 88.4% [3].

• ***SVT Criteria applied to faster rates: ***The SVT limit and VF rate cut-off will be increased to 200 bpm in all patients to provide SVT discrimination at faster rates. Two of the top five reasons for inappropriate detections in the GEM DR Study (933 patients) were a ventricular rate during AF in VF zone and a SVT cycle length faster than programmed SVT limit [3].

• ***Avoid detecting 1:1 SVTs with Long PRs as VT: ***1:1 SVTs with long PR intervals accounted for 38% of inappropriate detections in the Gem DR (7271) Clinical Study [3]. A retrospective analysis found that changing the 1:1 VT-ST boundary programmable parameter from 50% to 66% might eliminate 32% of all inappropriate detections. The downside to this approach is that it may result in a 0.8% rate of VT/VF misclassification or delay [21].

• ***Longer detection duration: ***VF initial beats to detect will be set to 18 of 24. Shorter beats to detect may result in more unnecessary shocks for SVTs or ventricular over-sensing.

• ***ATP attempts: ***In addition to terminating ventricular arrhythmias without shocks, ATP should eliminate some inappropriate shocks when inappropriate detections occur by terminating SVTs or slowing conduction.

The ***VT rate cut-off ***is one of the most important ICD settings because it can result in untreated symptomatic VT if set too fast, however it can result in unnecessary therapies for non-sustained VT, SVTs, or sensing issues, if set too slow. Reports have shown that some secondary prevention patients have significant symptoms for VTs outside treated zones [22]. The VT cut-off in the empiric arm is set to ≤ 150 bpm to err on the side of treating VTs and to advance the understanding of the incidence of slower VTs in all patient populations. The optimal VT rate cut-off may need to be set according to the patient's presenting conditions at implant (e.g., faster cut-off in primary prevention patients).

## Statistical Considerations

The primary endpoint is the proportion of true episodes that are shocked during the 12-month follow-up period. The standardized empiric programming regimen will be considered non-inferior to the physician tailored programming approach if *both *the proportion of shocked VT/VF episodes *and *the proportion of shocked SVT episodes are no more than 10 percentage points greater in the empiric arm than the physician tailored arm. The chosen margin 10 percent is considered clinically important.

It is assumed that 24% of patients will have at least one true VT/VF episode and 33% of patients will have at least one true SVT episode during the 12-month follow-up period. Based on unpublished data from other Medtronic trials, the within-patient correlation coefficient for multiple episodes is assumed to be 0.3. Assuming a similar distribution of episode counts per patient as observed in these previous trials and a shock rate of 30% and 14% for VT/VF and SVT episodes respectively, a total of 900 patients (450 in each arm) will give at least 80% power for the VT/VF hypothesis and 90% power for the SVT hypothesis, each tested at the significance level 0.05.

The critical secondary endpoint, time to first shock therapy, will be analyzed using the Cox proportional hazards model for 1) any VT/VF or SVT, 2) true VT/VF only and 3) true SVT only. The empiric programming approach will be considered non-inferior if the upper confidence limit for the hazard ratio is less than 1.5.

### Other Planned Analyses

To better understand the changing ICD patient populations, we will investigate whether or not the proportion of appropriate and inappropriate shocks delivered is related to the following baseline characteristics: main indication for implant (especially spontaneous sustained monomorphic VT), left ventricular ejection fraction, CAD status, history of Atrial Tach/Atrial Fib/Atrial Flutter, NYHA classification, use of amiodarone, sotalol, or beta-blockers, and inducibility for VT/VF. In addition, to facilitate understanding of the optimal programmable settings for various patient sub-groups, we will consider the impact of programmable settings on outcomes. In particular, we will examine the "treated cut-off" (TC), which is the VT detection cut-off if VT detection is 'On' or the VF detection cut-off if VT detection is 'Off' or 'Monitor'. Outcomes in patients with a faster TC (physician tailored arm) will be compared to patients with slower TC (either physician tailored arm or empiric arm). Other programmable settings that will be investigated include the number of beats to detect VF and the number of ATP attempts based at various rates (e.g., <175 bpm, 175–200 bpm, >200 bpm). The types of arrhythmias, median ventricular cycle length, and therapies delivered will also be characterized relative to the patient's conditions and programming. Furthermore, the incidence of slower VTs in patients without a history of spontaneous, sustained monomorphic VT will be characterized.

## Conclusions and Trial Impact

The EMPIRIC trial is a worldwide, multi-center, prospective, one-to-one randomized comparison of shock- related morbidity in a population of about 900 ICD patients whose ICD therapy is determined either by a standardized programming regimen or by physician tailored programming of VT/VF detection and therapy. Shock-related morbidity is assessed by a primary objective that compares between study arms the proportion of VT/VF episodes that are shocked and the proportion of SVT episodes that are shocked, and by a key secondary endpoint that compares to time to first shock therapy.

ICD patient populations have rapidly changed within the last five years but little has been published on optimal programming for the emerging patient subsets (e.g., primary prevention). Therefore a standardized regimen of parameters is used in this trial for all patient populations. Today's patient population is quite diverse, so a slightly more sophisticated programming approach may be necessary (e.g. change VT cut-off based on main ICD indication) or perhaps complex physician tailoring is critical to reducing shocks.

The EMPIRIC trial will characterize the shock morbidity of a single empiric programming approach compared to patient-specific, physician tailored programming. Empiric programming may be an acceptable strategy if it achieves equivalence with physician tailored programming. The EMPIRIC trial results will also provide a better understanding of how particular programming strategies impact the frequency of shocks delivered and will facilitate a way to optimize complex ICD programming.

## Competing Interests

1. Have you received reimbursements, fees, funding, or salary from an organization that may in any way gain or lose financially from the publication of this paper in the past five years, or is such an organization financing the article-processing charge for this article?

Dr. Morgan: Yes, Medtronic has paid me honoraria.

Dr. Sterns: Yes, I am a paid investigator in several Medtronic clinical trials and key investigator in the present trial. I understand that Medtronic is paying for the processing fee for this article.

Dr. Wilkoff: Yes, Medtronic, Guidant, St. Jude Medical

Hanson, Ousdigian, and Otterness: Yes, Employees of Medtronic.

2. Have you held any stocks or shares in an organization that may in any way gain or lose financially from the publication of this paper?

Dr. Morgan and Dr. Sterns and Dr. Wilkoff: No

Hanson, Ousdigian, and Otterness: Yes, own Medtronic stock.

3. Do you have any other financial competing interests?

Dr. Morgan and Dr. Sterns and Dr. Wilkoff: and Hanson and Ousdigian and Otterness: No.

4. Are there any non-financial competing interests you would like to declare in relation to this paper?

Dr. Morgan and Dr. Sterns and Dr. Wilkoff: and Hanson and Ousdigian and Otterness: No.

## Authors' Contributions

All 6 authors contributed to the study design and writing of this manuscript.
